# Intimate partner violence and timely antenatal care visits in sub-Saharan Africa

**DOI:** 10.1186/s13690-022-00853-y

**Published:** 2022-04-20

**Authors:** Richard Gyan Aboagye, Abdul-Aziz Seidu, Bernard Yeboah-Asiamah Asare, Collins Adu, Bright Opoku Ahinkorah

**Affiliations:** 1 Department of Family and Community Health, School of Public Health, University of Health and Allied Sciences, Hohoe, Ghana; 2 Centre for Gender and Advocacy, Takoradi Technical University, Takoradi, Ghana; 3 College of Public Health, Medical and Veterinary Sciences, James Cook University, Townsville, Australia; 4 Faculty of Built and Natural Environment, Department of Real Estate Management, Takoradi Technical University, Takoradi, Ghana; 5 Curtin School of Population Health, Curtin University, Perth, Australia; 6 Institute of Applied Health Sciences, University of Aberdeen, Aberdeen, Scotland, UK; 7 Department of Health Promotion, Education and Disability Studies, Kwame Nkrumah University of Science and Technology, Kumasi, Ghana; 8 School of Public Health, Faculty of Health, University of Technology Sydney, Sydney, Australia

**Keywords:** Intimate partner violence, Timely antenatal care visits, Pregnant women, Sub-Saharan Africa

## Abstract

**Background:**

Intimate partner violence (IPV) during pregnancy has negative physical and psychological health consequences on the pregnant women. As such, women who experience IPV during pregnancy are likely to have challenges accessing maternal healthcare services. In this study, we examined the influence of exposure to IPV on timely antenatal care (ANC) visits in sub-Saharan Africa.

**Methods:**

Cross-sectional data from the most recent Demographic and Health Survey of twenty-two countries in sub-Saharan Africa between 2012 and 2020 were analysed. Data were obtained from 61,282 women with birth history in the five years prior to the survey. A multilevel logistic regression was used to determine the association between IPV and timely ANC visits while controlling for significant covariates. Adjusted odds ratios (aOR) with 95% Confidence Intervals (CI) were used to present results from the multilevel logistic regression analysis.

**Results:**

The prevalence of timely ANC visit and IPV were 38.1% and 34.9% respectively. The highest and lowest prevalence of IPV were found in Sierra Leone (52.9%) and Comoros (8.1%), respectively. Timely ANC attendance among pregnant women was more prevalent in Liberia (74.9%) and lowest in DR Congo (19.0%). Women who experienced IPV during pregnancy were less likely to utilize timely ANC (aOR = 0.89, 95% CI = 0.86–0.92) compared to those who did not experience IPV. In terms of the covariates, the odds of timely ANC were higher among women aged 40–44 compared to those aged 15–19 (aOR = 1.35, 95% CI = 1.21–1.51). Higher odds of timely ANC was found among women who were cohabiting (aOR = 1.15, 95% CI = 1.10–1.20), those from the richest wealth quintile (aOR = 1.38, 95% CI = 1.28–1.48), those exposed to watching television (aOR = 1.24, 95% CI = 1.18–1.30), and those with health insurance (aOR = 1.46, 95% CI = 1.37–1.56).

**Conclusion:**

Findings from the study indicate the role of IPV in timely ANC visit in sub-Saharan Africa. To enhance timely ANC visits, there is the need for policy makers to strengthen and enforce the implementation of policies that alleviate IPV during pregnancy. Education and sensitization of married and cohabiting women and men on the negative effects of IPV on timely ANC should be done using media sources such as television. Inequalities in timely ANC can be eliminated through the provision and strengthening of existing maternal health policies such as health insurance.

## Background

Antenatal care (ANC) is a form of preventive health care [1], and described by the World Health Organization (WHO) as care including screening for risk; prevention, diagnosis and treatment of diseases; and health education and promotion delivered by skilled healthcare personnel to pregnant women appropriate to guarantee that both mother and baby are in optimum health conditions throughout the gestation period [2]. ANC decreases maternal and perinatal morbidity and mortality [2] directly by identifying complications associated with pregnancy for treatment, and indirectly by detecting women at increased risk of developing complications to ensure the appropriate needed referrals and detecting other causes of maternal death such as malaria and HIV infections [2]. Yet, pregnancy-related deaths remain unacceptably high and a public health problem worldwide [3]. It was estimated in 2017 alone that about 295,000 women lost their lives during pregnancy and after delivery worldwide [3], and about 2 million stillbirths are also reported every year [4]. These deaths are disproportionately high in low-and middle-income countries, especially in sub-Saharan Africa (SSA) [3, 4].

WHO recommends a minimum of eight ANC attendance up from the previous minimum visits of four throughout pregnancy [2, 5]. It also suggests pregnant women to have timely ANC contacts/visits, with the first contact/visit taking place within the first 12 weeks of pregnancy and subsequent visits taking place at “20, 26, 30, 34, 36, 38 and 40 weeks” [5]. The timely and appropriate ANC practices are recommended during pregnancy in order to realize the optimum life-saving prospective of ANC for mothers and their babies [2]. ANC visits in a timely manner is usually indicated as a way to effectively avert adverse pregnancy related outcomes [6], as research have demonstrated that receiving the WHO recommended ANC services is associated with the timing of first ANC visit by pregnant women [7]. For instance, evidence have shown that first visit within the first trimester and/or at least four visits during pregnancy are associated with lower risk of neonatal mortality [8].

Though ANC has since seen increased attendance over the past decade [2, 5], its utilization is still suboptimal in low-and middle-income countries [2] with only 70.2% of pregnant women utilizing ANC for at least four times during the period 2015 to 2020 globally [1]. Predominately, sub-Saharan African countries report the lowest ANC utilization levels [1]. Studies have identified socio-demographic factors including maternal age, parity, education, socioeconomic status, place of residence; health facility factors such as quality of care, distance to health care facility, having insurance cover, and cost of ANC services; and psychosocial factors such as exposure to the media, partner’s support, and attitude towards ANC utilisation [6, 9–18] as predictors of the utilization of ANC and its timeliness.

Gender-based violence including intimate partner violence (IPV) during pregnancy has been shown to be a significant risk for complications during childbirth, as such pregnant women who are exposed to IPV are required to be identified during ANC [19]. IPV is a global public health problem, with an estimated 27% [Uncertain Interval (UI) 23–31%] of women aged 15–49 experiencing physical and/or sexual violence perpetrated by an intimate partner [20], and SSA is one of the regions around the world to report the highest prevalence (33%) of IPV [20]. IPV is suggested to be a sign of disempowering women in taking part in decision making in the house, controlling economic resources, and sexual consent which have been suggested to enable women to have better health outcomes [21]. Perpetrators of IPV are also found to exhibit controlling behaviors such as restriction of partner’s movement and relationships with family and friends [22, 23]. These may typically limit pregnant women exposed to IPV from accessing ANC. Studies have found IPV to be associated with reduced ANC utilization in several low-and middle-income countries [24–29]. For instance, in a meta-analysis by Musa et al. [26], experiencing IPV was found to be associated with 25% decrease in the use of adequate ANC among women.

Previous studies in SSA have examined the influence of experiencing IPV on several indicators of maternal health care service utilization such as skilled delivery attendance [21, 30] and adequate use of ANC (attendance of 4 or more) [24, 25, 31–33].

However, limited studies have focused on the effect of exposure to IPV on the timely use of ANC (e.g., first visit in the 12 weeks of pregnancy) among pregnant women in sub-Saharan African countries [27]. Using nationally representative surveys from several sub-Saharan African countries, we examined the association between exposure to IPV and timely utilization of ANC in SSA. Providing a comprehensive analysis across several sub-Saharan African countries could help clarify and highlight IPV as a risk factor for timely ANC visits.

## Methods

### Data source and study design

We combined data from the most recent Demographic and Health Survey (DHS) of 22 countries in SSA with a dataset between 2012 and 2020 for this study (Table 1). We included countries with observations on the domestic violence module as well as the other variables considered for this study. The data were extracted from the individual recode (IR) files in the countries' datasets. The DHS is a globally comparable nationally representative survey that was undertaken in a number of low- and middle-income countries [34]. The DHS used a structured questionnaire to collect data from respondents in a cross-sectional manner. Domestic violence, maternal health care service utilisation, mother and child health, reproductive health, and men's health are among the topics explored in the DHS [34]. The DHS used a two-stage cluster sampling method to sample the respondents. Initially, clusters were chosen, which were made up of enumeration areas (EA). A systematic sampling of households was performed in the second step. In each of the selected EAs, a household listing operation was conducted, and households to be included in the survey were chosen at random from the list. The survey methodology has been discussed in a study by Aliaga and Ruilin [35]. We included 61,282 women with children in the five years before the surveys who had complete cases on all variables of interest. We relied on the Strengthening Reporting of Observational studies in Epidemiology (STROBE) reporting guidelines in drafting this paper [36]. The dataset is freely accessible via this link: https://dhsprogram.com/data/available-datasets.cfm.

**Table 1:** Description of sample

Country	Year of survey	Weighted N	Weighted %
1.Angola	2015–16	3,824	6.2
2.Benin	2018	2,725	4.4
3.Burundi	2016–17	5,424	8.9
4.DR Congo	2013–14	3,342	5.4
5.Cameroon	2018	2,556	4.2
6.Ethiopia	2016	1,904	3.1
7.Gabon	2012	1,557	2.5
8.Gambia	2019–20	1,033	1.7
9.Kenya	2014	2,265	3.7
10.Comoros	2012–13	1,314	2.1
11.Liberia	2019–20	1,040	1.7
12.Mali	2018	1,888	3.1
13.Malawi	2015–16	3,262	5.3
14.Nigeria	2018	5,059	8.3
15.Namibia	2013	588	1.0
16.Sierra Leone	2019	2,331	3.8
17.Chad	2014–15	1,451	2.4
18.Togo	2013–14	3,130	5.1
19.Tanzania	2015–16	4,517	7.4
20.Uganda	2016	4,500	7.3
21.Zambia	2018	4,351	7.1
22.Zimbabwe	2015	3221	5.3
**All countries**		**61,282**	**100.0**

### Study variables

#### Outcome variable

Timing of ANC was the outcome variable. It was assessed using the question “*How many months pregnant were you when you first received ANC for this pregnancy?*”. Women who attended their first ANC within the first 3 months of pregnancy ( ≤ 3 months) were classified as having "timely ANC visit" whilst the remaining pregnant women were grouped as having "late ANC visit". This categorisation was informed by previous studies [37–40].

#### Key explanatory variable

The key explanatory variable was IPV. The variable was assessed using three other variables namely physical violence, emotional violence, and sexual violence. Questions from the modified version of the conflict tactics scale were used to assess these three variables [41, 42]. Details of the questions, responses to the questions, and their categorization have been used in previous studies [43, 44].

#### Covariates

Based on review of literature on determinants of timely ANC visits [37–40] and variables available in the DHS, we included age of the women and their partners, educational level of the women and their partners, marital status, current working status of the women, exposure to radio, exposure to television, exposure to newspapers/magazine, parity, national health insurance subscription, wealth quintile, place of residence and geographical subregions as covariates. These variables were further grouped into individual level and contextual level variables.

#### Individual level factors

We utilised the existing coding for maternal age, educational level of the women and their partners, current working status, and health insurance subscription in the DHS dataset. Marital status was recoded as “married” and “cohabiting”. Parity was recoded into “1”, “2”, “3”, and “4 or more”. Partner’s age was recoded as “15–24”, “25–34”, “35–44”, and “45 + ”. Exposure to listening to radio (no and yes), exposure to watching television (no and yes), and exposure to reading newspapers/magazines (no and yes) were generated from responses to questions on frequency of reading newspapers, frequency of listening to radio, and frequency of watching television.

#### Contextual level factors

The contextual level variables consisted of place of residence, wealth quintile, and geographical subregions. The coding for wealth quintile and place of residence as found in the DHS were maintained and used in the final analysis. With geographical sub-regions, we grouped the countries included in the study into "Eastern", "Central", "Western", and "Southern" Africa respectively based on their location in SSA.

### Statistical analyses

The data analysis was carried out using Stata software version 16.0 (Stata Corporation, College Station, TX, USA). We used percentages to summarise the prevalence of timely ANC and IPV in SSA. To examine the distribution of timely ANC across IPV and the covariates, we employed cross-tabulation (Table 2). Pearson chi-square test of independence was used to determine the variables that were significantly associated with timely ANC (Table 2). In addition, we used four models in a multilevel binary logistic regression analysis to investigate the association between IPV and timely ANC while adjusting for the individual and contextual level variables (Table 3). Model 0 demonstrated how the clusters within the primary sampling units (PSUs), contributed to the variance in timely ANC. IPV and the individual level variables were incorporated into Model I. The contextual level covariates were included in Model II. Finally, IPV, individual-level, and contextual level variables were included in Model III. In order to fit the four models, we used the Stata command "melogit." We included Akaike's Information Criterion (AIC) tests for model comparison. The adjusted odds ratios (aOR) with 95 percent confidence intervals (CIs) were used to present the results of the multilevel binary logistic regression. Sample weights were applied in the analysis to deal with under-sampling and over-sampling and correction for the complex survey design was addressed.

**Table 2:** Bivariate analysis of intimate partner violence and timely antenatal care visits in sub-Saharan Africa

**Variable**	**Weighted N**	**Weighted %**	**Timely ANC**		
			**No (%)**	**Yes (%)**	***P-value***
**Intimate partner violence**					< 0.001
No	39,866	65.1	60.5	39.5	
Yes	21,416	34.9	64.5	35.5	
**Maternal age**					0.012
15–19	3,188	5.2	64.7	35.3	
20–24	13,315	21.7	61.7	38.3	
25–29	17,031	27.8	61.4	38.6	
30–34	13,733	22.4	61.5	38.5	
35–39	8,896	14.5	61.7	38.3	
40–44	3,947	6.5	62.2	37.8	
45–49	1,172	1.9	66.7	33.3	
**Maternal educational level**					< 0.001
No education	17,190	28.0	62.9	37.1	
Primary	23,991	39.2	65.8	34.2	
Secondary	17,402	28.4	57.8	42.2	
Higher	2,699	4.4	46.0	54.0	
**Marital status**					< 0.001
Married	47,262	77.1	62.6	37.4	
Cohabiting	14,020	22.9	59.3	40.7	
**Current working status**					0.271
No	19,684	32.1	61.4	38.6	
Yes	41,598	67.9	62.1	37.9	
**Exposure to listening to radio**					< 0.001
No	38,090	62.2	63.0	37.0	
Yes	23,192	37.8	60.1	39.9	
**Exposure to watching television**					< 0.001
No	44,429	72.5	65.3	34.7	
Yes	16,853	27.5	52.8	47.2	
**Exposure to reading newspaper/magazine**					< 0.001
No	56,789	92.7	62.7	37.3	
Yes	4,493	7.3	51.1	48.9	
**Partner’s age**					0.123
15–24	4,027	6.6	63.5	36.5	
25–34	24,500	40.0	61.5	38.5	
35–44	22,010	35.9	61.6	38.4	
45 +	10,745	17.5	62.5	37.5	
**Partner’s educational level**					< 0.001
No education	13,155	21.5	62.2	37.8	
Primary	21,145	34.5	66.2	33.8	
Secondary	21,775	35.5	60.2	39.8	
Higher	5,207	8.5	50.5	49.5	
**Parity**					< 0.001
1	10,600	17.3	57.6	42.4	
2	12,306	20.1	59.8	40.2	
3	11,002	17.9	60.6	39.4	
4 or more	27,374	44.7	65.0	35.0	
** Health insurance coverage**					< 0.001
No	56,846	92.8	62.8	37.2	
Yes	4,436	7.2	50.3	49.7	
**Wealth quintile**					< 0.001
Poorest	12,105	19.8	66.0	34.0	
Poorer	12,669	20.7	65.1	34.9	
Middle	12,626	20.6	64.5	35.5	
Richer	12,471	20.3	61.6	38.4	
Richest	11,411	18.6	51.2	48.8	
**Place of residence**					< 0.001
Urban	21,704	35.4	56.6	43.4	
Rural	39,578	64.6	64.8	35.2	
**Sub-regions**					< 0.001
Southern	588	1.0	55.1	44.9	
Eastern	30,757	50.2	65.0	35.0	
Central	12,731	20.8	58.2	41.8	
Western	17,206	28.1	59.3	40.7

**Table 3:** Fixed and random effects analysis of the association between intimate partner violence and timely antenatal care visits in sub-Saharan Africa

Variables	Model O	Model I aOR [95% CI]	Model II aOR [95% CI]	Model III aOR [95% CI]
**Fixed effects**				
**Intimate partner violence**				
No		Reference		Reference
Yes		0.89^***^ [0.86,0.92]		0.89^***^ [0.86,0.92]
**Maternal age**				
15–19		Reference		Reference
20–24		1.14^**^ [1.05,1.24]		1.14^**^ [1.05,1.24]
25–29		1.25^***^ [1.14,1.37]		1.22^***^ [1.12,1.33]
30–34		1.34^***^ [1.22,1.47]		1.29^***^ [1.17,1.42]
35–39		1.36^***^ [1.23,1.50]		1.30^***^ [1.17,1.44]
40–44		1.40^***^ [1.25,1.57]		1.35^***^ [1.21,1.51]
45–49		1.21^*^ [1.04,1.41]		1.16^*^ [1.00,1.36]
**Maternal educational level**				
No education		Reference		Reference
Primary		0.89^***^ [0.85,0.93]		0.93^**^ [0.89,0.98]
Secondary		0.99 [0.94,1.05]		0.99 [0.94,1.05]
Higher		1.13^*^ [1.02,1.26]		1.07 [0.96,1.20]
**Marital status**				
Married		Reference		Reference
Cohabiting		1.13^***^ [1.09,1.18]		1.15^***^ [1.10,1.20]
**Partner’s educational level**				
No education		Reference		Reference
Primary		0.84^***^ [0.80,0.89]		0.88^***^ [0.84,0.93]
Secondary		0.93^**^ [0.88,0.98]		0.93^**^ [0.88,0.98]
Higher		1.07 [0.98,1.16]		1.01 [0.92,1.09]
**Parity**				
1		Reference		Reference
2		0.87^***^ [0.82,0.92]		0.87^***^ [0.83,0.93]
3		0.83^***^ [0.78,0.88]		0.84^***^ [0.79,0.89]
4 or more		0.68^***^ [0.64,0.73]		0.70^***^ [0.66,0.75]
**Exposure to listening to radio**				
No		Reference		Reference
Yes		0.97 [0.94,1.01]		0.97 [0.93,1.01]
**Exposure to watching television**				
No		Reference		Reference
Yes		1.37^***^ [1.31,1.43]		1.24^***^ [1.18,1.30]
**Exposure to reading newspaper/magazine**				
No		Reference		Reference
Yes		1.10^**^ [1.03,1.18]		1.12^**^ [1.05,1.21]
**Health insurance coverage**				
No		Reference		Reference
Yes		1.45^***^ [1.36,1.54]		1.46^***^ [1.37,1.56]
**Wealth quintile**				
Poorest			Reference	Reference
Poorer			1.04 [0.99,1.10]	1.04 [0.99,1.10]
Middle			1.00 [0.95,1.06]	0.98 [0.93,1.04]
Richer			1.14^***^ [1.08,1.21]	1.05 [0.99,1.12]
Richest			1.74^***^ [1.63,1.85]	1.38^***^ [1.28,1.48]
**Place of residence**				
Urban			Reference	Reference
Rural			0.93^**^ [0.89,0.98]	1.01 [0.97,1.06]
**Sub-regions**				
Southern			Reference	Reference
Eastern			0.74^***^ [0.63,0.87]	0.89 [0.76,1.05]
Central			0.90 [0.77,1.06]	1.00 [0.85,1.18]
Western			0.96 [0.81,1.12]	1.13 [0.95,1.33]
**Random effects**				
Primary Sampling Unit variance (95% CI)	0.05 [0.04, 0.07]	0.04 [0.03, 0.06]	0.05 [0.04, 0.06]	0.05 [0.03, 0.06]
Intracluster correlation coefficient	0.01	0.01	0.01	0.01
Likelihood ratio Test	165.93 (< 0.001)	124.55 (< 0.001)	144.12 (< 0.001)	123.06 (< 0.001)
Wald chi-square	Reference	1250.11***	867.21***	1475.59***
**Model fitness**				
Log-likelihood	-40,584.608	-39,946.864	-40,148.688	-39,828.439
Akaike’s Information Criterion	81,173.22	79,939.73	80,317.38	79,718.88
Sample size	61,282	61,282	61,282	61,282
Number of clusters	1,569	1,569	1,569	1,569

Exponentiated coefficients; 95% confidence intervals in brackets; *aOR* adjusted odds ratios;* CI* Confidence Interval; ^*^ *p* < 0.05, ^**^ *p* < 0.01, ^***^ *p* < 0.001

## Results

### Prevalence of intimate partner violence and timely antenatal care visit in sub-Saharan Africa

**Fig. 1:**
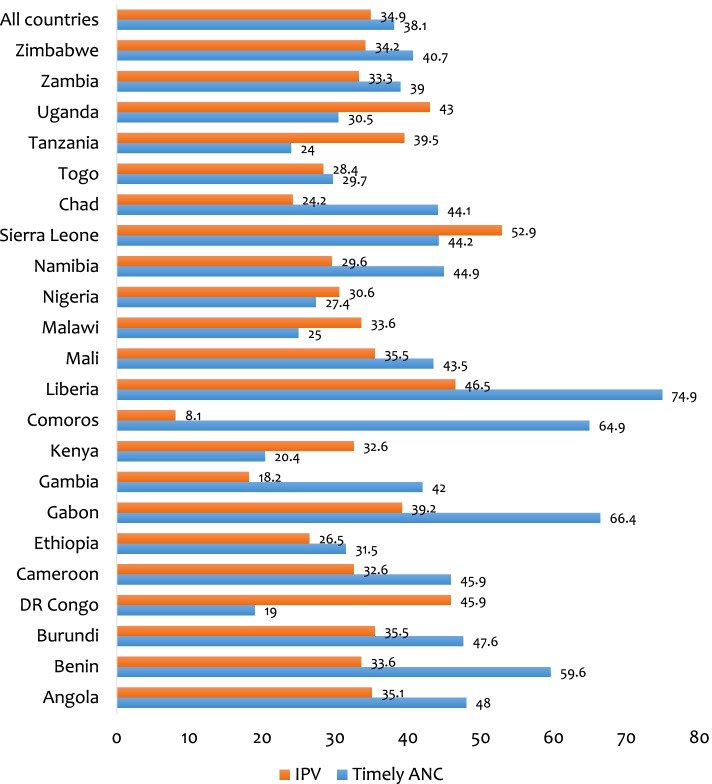
Prevalence of intimate partner violence and timely ANC attendance among pregnant women in sub-Saharan Africa

Figure 1 depicts the prevalence of IPV and timely ANC attendance in SSA. In this study, the pooled prevalence of timely ANC visits and IPV was 38.1 percent and 34.9 percent, respectively. Sierra Leone had the highest prevalence of IPV (52.9%), while Comoros had the lowest (8.1%). Pregnant women's timely ANC attendance was highest in Liberia (74.9%) and lowest in DR Congo (19.0%).

### Distribution of timely ANC visit across intimate partner violence and covariates

Table 2 shows the bivariate analysis of IPV and timely ANC visit in SSA. The results showed significant association between IPV and timely ANC visit. The prevalence of timely ANC visit was higher (38.6%; *p* < 0.001) among women aged 25–29 compared with those aged 45–49 (33.3%; *p* = 0.01). Cohabiting women recorded a higher proportion of timely ANC compared to married women (40.7%; < 0.001). Furthermore, there were higher proportions of timely ANC among women who were exposed to listening to radio (39.9%; < 0.001), exposed to watching television (47.2%; < 0.001), and exposed to reading newspapers (48.9%; < 0.001) compared to those who were not exposed. The results showed a higher proportion of timely ANC visits among women who were covered by national health insurance (49.7%; < 0.001), from Southern region (44.9%; < 0.001), with one child (42.4%; < 0.001), from urban centers (43.4%; < 0.001), and within richest wealth quintile (48.8%; < 0.001).

### Association between IPV and timely ANC attendance among women in sub-Saharan Africa

Table 3 shows the fixed and random effects analysis of the association between IPV and timely ANC in SSA. Women who experienced IPV were less likely to utilize ANC timely (aOR = 0.89, 95% CI = 0.86–0.92) compared to those who were not exposed to IPV. In terms of the covariates, the odds of timely ANC were higher among women aged 40–44 compared to those aged 15–19 (aOR = 1.35, 95% CI = 1.21–1.51). Higher odds of timely ANC was found among women who were cohabiting (aOR = 1.15, 95% CI = 1.10–1.20), those of the richest wealth quintile (aOR = 1.38, 95% CI = 1.28–1.48), those exposed to television (aOR = 1.24, 95% CI = 1.18–1.30), and those on NHIS (aOR = 1.46, 95% CI = 1.37–1.56) compared to their counterparts.

## Discussion

The influence of IPV on timely ANC attendance in SSA was investigated in this study. We found the prevalence of IPV and timely ANC to be 34.9 percent and 38.1 percent, respectively. The current prevalence of timely ANC attendance is higher than what was found in previous studies in Ethiopia (13.2%-17.4%) [45], Nigeria (15.4%) [46], Zambia (17%) [47], and Tanzania (12.4%) [48]. The possibility of high rate of timely ANC attendance could be due to the various interventions and policies such as national health insurance scheme and free maternal healthcare service implemented within the sub-region [49–51]. The prevalence of IPV is higher than what was found in a previous study on polygyny and IPV in 16 countries in SSA [52]. The difference in prevalence could be attributed to differences in sample sizes, geographical scope, and study periods.

 Women in SSA who experienced IPV were less likely to utilize timely ANC compared to those who did not experience IPV. This finding is consistent with previous studies conducted in Benin [53], Ethiopia [54], Mozambique [55], Nigeria [33], and Pakistan [56]. The possible justification could be due to the lack of social support from their partners/husbands to inform them on their timely utilization of ANC. Therefore, women at their gestation age less than 12 weeks might not develop a positive behavioral change towards timely ANC attendance. Also, the association between IPV and timely ANC can be explained by the fact that violence can have a negative influence on a woman's mental and physical health, making it difficult for her to seek good maternal health care. The result in the present study emphasizes the need for healthcare professionals to provide counselling and trauma management services to pregnant women experiencing IPV. In view of this, the involvement and empowerment of women in decision making is crucial towards improving maternal health service utilization as found in previous studies [57, 58]. However, previous studies in Tanzania and Rwanda found no association between IPV and timely ANC visit [59, 60]. 

 Women with a parity of four or more were less likely than those with a parity of one to begin early ANC. This finding is consistent with earlier research [61, 62], which identified high parity to be associated with later commencement of ANC attendance. The possible explanation is that women with high parity may consider themselves as experienced due to their multiple successful pregnancies and childbirth, and hence may postpone ANC commencement [63]. For women and their unborn children, starting ANC visits on time is crucial for disease prevention, health promotion, and curative therapy [12]. According to the research, decreased ANC use among high-parity women could be owing to a lack of time available for ANC attendance due to child care, unpleasant ANC experiences from previous pregnancies, and insufficient family resources [64].

Previous study has shown that being married has a protective effect on ANC use. In this study, however, cohabiting pregnant women were more likely than married pregnant women to start ANC on time. This result contradicts the findings of Okedo-Alex et al. [12]. According to Okedo-Alex et al. [12], married pregnant women receive financial and psychosocial assistance from their spouses, and societal acceptance and support for their pregnancy condition prompts them to seek ANC as soon as possible. Unmarried women were also more likely to use ANC services, according to Tarekegn, Lieberman, and Giedraistis [65]. 

Poverty is a known impediment to healthcare utilisation in SSA, and low-income women may be unable to pay the costs of using ANC [53]. Our data demonstrated that in SSA, the health insurance and wealth level were significant factors impacting timely ANC utilisation among pregnant women. Pregnant women in the greatest wealth quintile and those covered by the health insurance were more likely to start ANC on time. This result is consistent with prior studies [12, 53]. Pregnant women may begin ANC late in pregnancy because to a lack of financial resources. Previous research has shown that socioeconomic status has an impact on timely ANC usage [49, 64, 66]. Although some countries in SSA, such as Ghana, have a national policy requiring all pregnant women to enroll in the national health insurance scheme for free maternal health services, pregnant women in some countries in SSA may still pay out of pocket for some direct medical costs associated with maternal healthcare. These expenses make it difficult for pregnant women to access ANC services on time [50, 51]. As a result, maternal health services, such as ANC, should be offered free through required social health insurance to enhance utilisation and, as a result, reduce maternal death and morbidity.

### Strengths and limitations

We employed nationally representative data from 22 countries in SSA, making our findings generalizable to married and cohabiting women in the countries considered. Another strength is the use of large datasets with large sample sizes and the robust statistical analysis performed. However, the study design employed by the DHS is cross-sectional and this makes it impossible to establish any causal inferences. Again, there is the likelihood of variations in relationships between IPV and timely ANC since the survey years for the surveys varied. Finally, recall and social desirability bias may affect the reporting of the prevalences of IPV and timely ANC visits.

## Conclusion

Findings from the study indicate the role of IPV in timely ANC visit in SSA. To enhance timely ANC visits, there is the need for policy makers to strengthen and enforce the implementation of policies that alleviate IPV. Education and sensitization of married and cohabiting women and men on the negative effects of IPV on timely ANC should be done using media sources such as television. Inequalities in timely ANC can be eliminated through the provision of free maternal health policies such as health insurance. 

## Data Availability

The datasets used and/or analysed during the current study are available from the corresponding author on reasonable request.

## Abbreviations

ANC: Antenatal care
IPV: Intimate partner violence
WHO: World Health Organization
SSA: Sub-Saharan Africa
DHS: Demographic and health survey
